# Supplementary material for: Enabling Coarse X-ray Fluorescence Imaging Scans with Enlarged Synchrotron Beam by Means of Mosaic Crystal Defocusing Optics
Source: Int J Mol Sci. 2022 Apr 23;23(9):4673. doi: 10.3390/ijms23094673 (PMC9104365; doi:10.3390/ijms23094673)
Supplement: Supplementary file 1 [file ijms-23-04673-s001.zip › ijms-1687089-supplementary.pdf]

# - Supplemental material –

Enabling coarse X-ray fluorescence imaging scans with enlarged synchrotron beam by means of mosaic crystal defocusing optics

Jonas Baumann[1,*], Christian Körnig[2], Theresa Staufer[2], Christopher Schlesiger[1], Oliver Schmutzler[2], Florian Grüner[2], Wolfgang Malzer[1] and Birgit Kanngießer[1]

1 TU Berlin, Analytical X-ray Physics, Hardenbergstr. 36, 10623 Berlin, Germany
2 Fachbereich Physik, Universität Hamburg and Center for Free-Electron Laser Science (CFEL), Luruper Chaussee 149, 22761 Hamburg, Germany
* Correspondence: jonas.baumann@physik.tu-berlin.de

S1. Reflectivity of cone and cylindrically shaped substrate

Since a cylindrically shaped substrate is more easily purchased than a cone shaped substrate, ray tracing simulations are performed to compare the performance of both optics. 5 million photons for the ray tracing are generated as defined in Section S2 to match the beamline parameters closely. The middle of the generated X-ray beam hits the cone at a radius of 10 mm. The radius of the cylinder optics is also 10 mm. Both optics are aligned such that the Bragg condition is fulfilled. As crystal, 100 µm of HAPG with a mosaic spread of 0.1° and a Lorentzian mosaicity is used. In Figure S1 a) the reflected intensity distribution on a detector 1500 mm away from the optics hit position is shown for the cone optics (left) and cylinder optics (right). The given reflectivity *R* is the ratio of the number of detected photons and generated photons. As can be seen, the overall reflectivity is almost the same for both optics. Only the shape slightly differs. Namely, the reflection profile of the cylindrically shaped optics is slightly larger and the top and bottom edges are less tilted. This might even be slightly advantageous for usage in a coarse scan. Interestingly, even for lower energies and thus larger Bragg angles, reflectivities for both optics are very similar as is indicated in Figure S1 b).

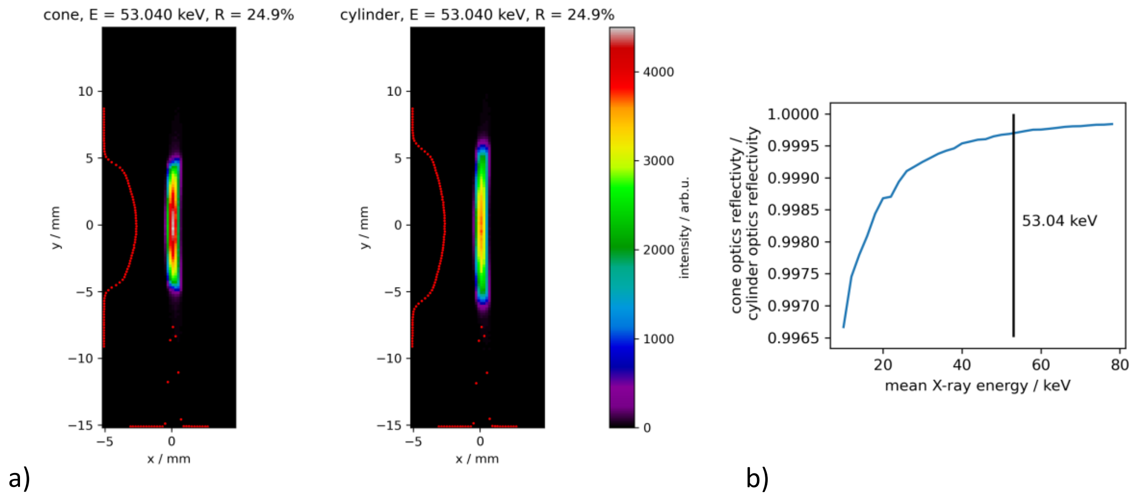

Figure S1: a) comparison of simulated reflection profile of a cone optics (left) and cylinder optics (right). b) reflectivity behavior compared for both optics for different photon energies.

S2. Ray tracing adaption for the direct X-ray beam

Figure S2 shows measurement and ray tracing results of the direct beam (53.04 keV) of beamline P21.1 at the synchrotron radiation facility PETRA III in Hamburg. The ray tracing simulation of the X-ray source was defined as described in the paper. To match the simulation to the measured profile, the distance and the widths of the two Gaussians defining the position distributions of the photons were adapted. As matching criterion, the full width at half maximum (FWHM) in Figure S2 g)-j) was used. The manual adjustment leads to an agreement of FWHM values for the two distances of better than 0.5%. While the overall size and shape fit well, small discrepancies are visible in Figures S2 e) and f) concerning the intensity distribution, which is more homogeneous but slightly asymmetric in the shown 'X-ray Eye' (Figure S2 e) measurement as compared to the ray tracing simulation (Figure S2 f).

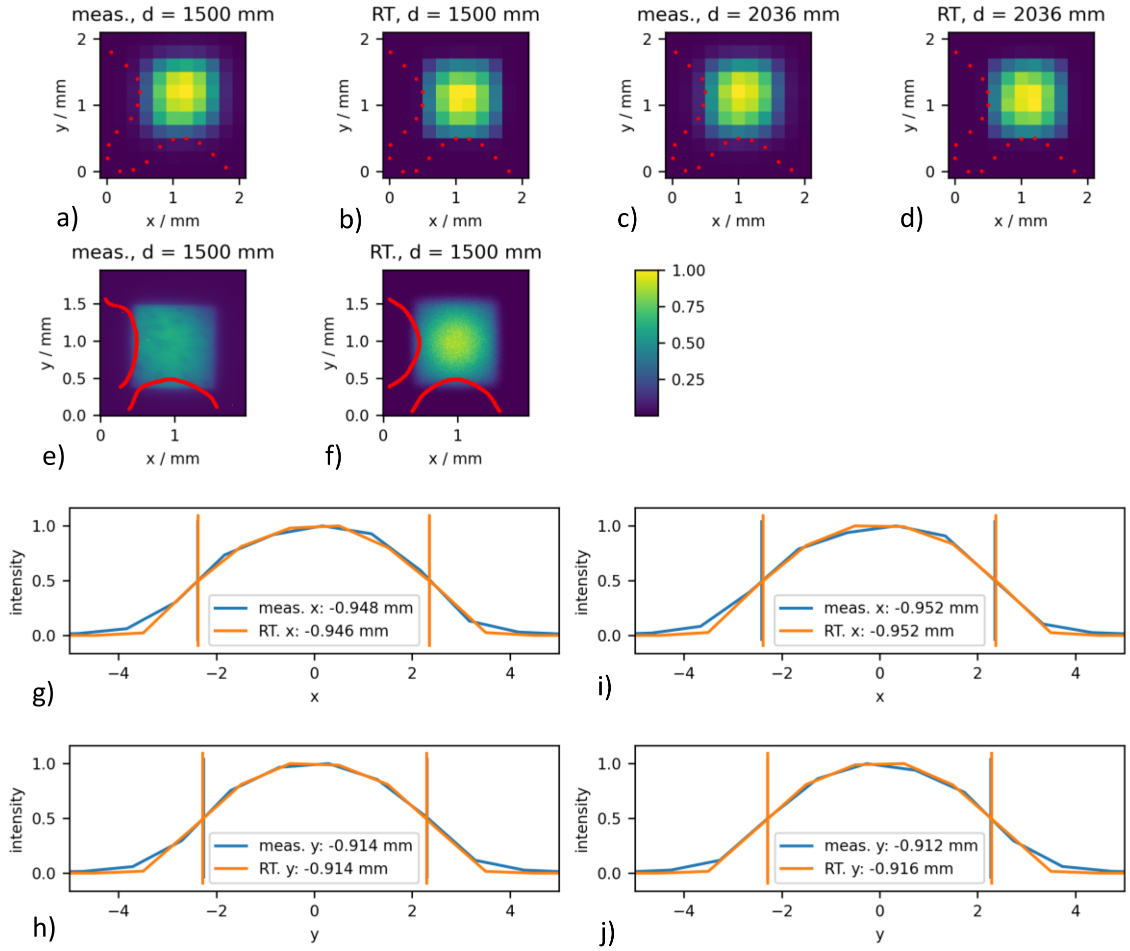

Figure S2: a) – d) measured ("meas.") and simulated ("RT") normalized intensity profile of the direct synchrotron beam at two different distances *d* from the optics as detected by the 'PE' detector. e) – f) As a) and b) but measured by and simulated for the 'X-ray Eye' detector. g) and h) show the column- and row-wise summed intensity profiles of a) and b). i) and j) show similar plots for the profiles of larger distances in c) and d). The full widths of half maximum are also given for images g) – j).

S3. Rocking curve measurements at 8.05 keV

Rocking curve measurements of the four optics and a 100 µm thick HAPG crystal on a flat glass substrate were performed with a D8 ADVANCE X-ray diffractometer (BRUKER) equipped with a Cu anode. The incoming beam is confined by a pinhole with 0.5 mm diameter. In front of the detector, a slit with a height of 0.6 mm is used. With a Bragg angle of 13.28°, the footprint on the crystal has a length of 2.2 mm.

Figure S3 shows the recorded rocking curves for all 4 crystals and the flat crystal in comparison. Clearly, the cylinder optics render a much larger and more inhomogeneous width, in accordance to the synchrotron measurements. For different positions on the optics along the cylinder axis, the rocking curves show strong deviations in shape, width and intensity (Figure S4).

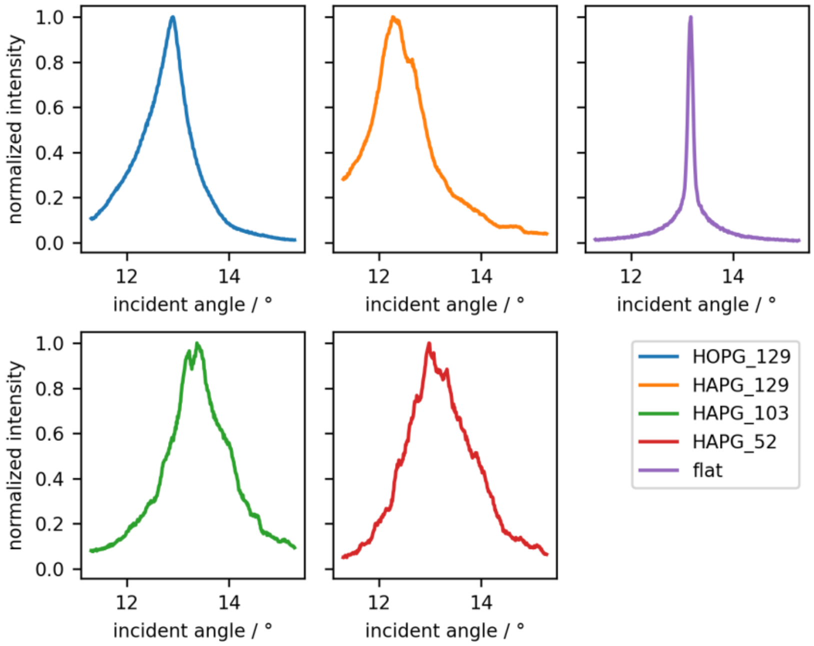

Figure S3: Comparison of the normalized laboratory rocking curve measurements for the four optics and a similar HAPG crystal applied to a flat glass substrate ('flat').

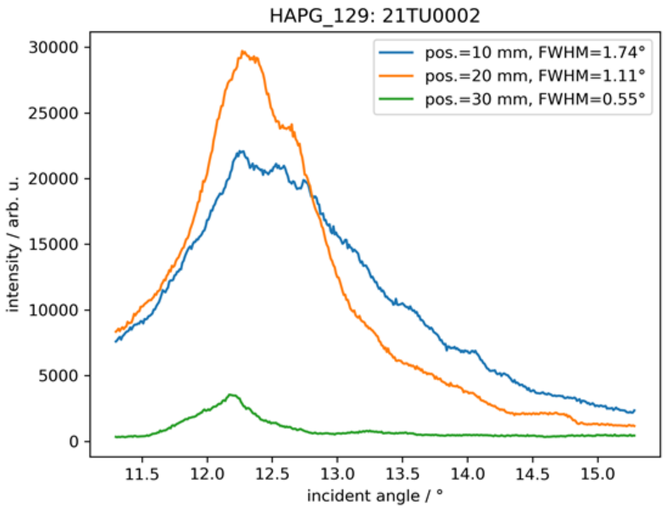

Figure S4: Rocking curves of HAPG_129 on different crystal positions along the cylinder axis.
